# Pseudokousma

**Published:** 1905-12

**Authors:** Frank H. Koyle

**Affiliations:** Hornellsville, N. Y.


### Pseudokousma

By DR. FRANK H. KOYLE, of Hornellsville, N. Y.

(Author's Abstract)

PSEUDOKOUSMA is that condition in which there is false perception of pitch. It is to be differentiated from diplacusis (in which a reduplication of the original note, or noise may be heard in one or both ears), a term almost invariably used indifferently to designate both "double-hearing" (diplacusis), and "false-hearing" (pseudokousma), as well as a composite condition including both phenomena. It is also to be differentiated from paracusis, which refers simply to an abnormal perception of sound. Among the authors who refer false-hearing to the internal ear, the writer mentions: Helmholtz, Benzold, Gellé, Gradenigo, Hans, Daae, Hensen, Hammerschlag, Von Wittich, Knapp, Spear, Gruber, Blau, Bonnier, Etrévant and Bishop. Blau and Bishop seem to be the only two who refer to a middle-ear cause exclusively without reference to a diplacusis. The theory of vonWittich, who claims that the perception of tone is brought about in the organ of Corti and that there the functional integrity of the endings of the nerve-fibres is affected as a result of altered labyrinthine pressure from exudation into the tympanic cavity, is claimed by the writer to have never been clinically demonstrated. Besides, if the fibres attuned to the tone B came into functional activity along with others corresponding to the tone A, the A fibres being also stimulated by the tone B, the resultant sound would be a discord, having no relation to overtones, harmonic or disharmonic.

The theory at one time advanced by Knapp that the lamina spiralis of one side was attuned for a different tone-scale than that of the other, has given away to the latter teachings of Helmholtz, who claims that the tone-perceiving function is to be referred to the membrana basilaris. The writer, in describing pseudokousma to the middle-ear causes, says: "In order to disprove the theory of a permanent structural lesion consisting of a faulty tuning, either of the fibres of Corti or the cords of the membrana basilaris as the etiological factor in this disease, it is only necessary to state that a disappearance of the pseudokousma has frequently resulted from treatment directed *solely to the middle- ear.* At the same time a functional derangement of the cochlear structures, co-existing and disappearing with lesions of the sound-conducting apparatus, is not to be denied. Whether the ultimate sound-sensation is ascribed to the fibres of Corti, or, as seems to be proven, to the cords of the membrana basilaris, we know that it is to external vibratory impulses that they respond, each being brought into sympathetic vibration with its fundamental, which, in turn, forms a part of the composite tone, or noise, from without. It follows, then, that since in this disease these cords do *not* reproduce the exact tone having an external origin, the character of the vibrations must have become altered *during transmission.*"

The writer briefly reviews the functions of the various tympanic structures concerned in audition, laying particular stress on the tympanic cavity and mastoid cells in their capacity as *resonance-chambers,* and shows how these functions are influenced by structural changes involving the sound-conducting (and auxiliary) apparatus. In support of his contention two cases are cited with details of the history and treatment of each, together with a series of tuning-fork experiments. In the *first* case, the immediate cause of the false-hearing is ascribed to a rupture of some, or all, of the fibres of the stapedius muscle, the loss of function of which, as a cause of tinnitus aurium, has been ably described (for the first time, so far as the writer has been able to learn) by Dr. Joseph Willets, of Pittsburg, Pa. In this (the writer's) case, the lesion was due to a sudden and powerful contraction of the muscle by galvanic electrical excitation, the patient, Dr. T., having attempted to relieve a temporary occlusion of both Eustachian tubes by placing the *anode* in his right and *cathode* in his left auditory canal. The writer's following summary will be sufficiently descriptive:

"In case I we have evidently a traumatic lesion from the unwise use of an unmeasured galvanic current which though described as *moderate,* was probably excessive. Prior to the *taking cold* there had been no conscious disability in this *left* ear; but suddenly, during the application of the electrical current, a *snap* is heard, described as being similar in quality and loudness to that of the "snapping of a tense violin string." *Immediately* there is a great *jangle* of sounds, but later this becomes modified and settles down to a constant tinnitus and a condition of false-hearing in the ear affected. There can be no doubt as to the cause of this profound disturbance of the labyrinthine contents. The clinical history of the case presents positive evidence of the partial or complete suspension of the force required to regulate the impact against the m. obturatoria. In other words, *the integrity of the stapedius muscle was impaired, or lost, when the snap occurred.* This is abundantly proven by the results of treatment directed toward the re-establishment of the normal plane of the m. tympani.

It has been shown that inflation of the tympanic cavity gave negative results, even augmenting the symtoms if any considerable pressure was used; while, on the other hand, the aspirating otoscope gave immediate relief from annoying subjective symptoms. The absence of intra-tympanic adhesions and spasm of the t. tympani muscle having been demonstrated by the results of catheterization, it was manifestly impossible that an augmentation of the subjective symptoms could have been due to any other cause than the absence of the *stapedian curb,* thus permitting an exaggerated pressure from without inwards, and a condensation of the intra-tympanic air, resulting in a profound disturbance of the labyrinthine elements. It is thus seen that as long as such inward pressure obtains, just so long will the *sound-conducting labyrinthine contents* continue to exercise a perverted function, and the floating terminal hair-cells and the basilaric fibres receive a false tone-image corresponding to the degree of compression exercised upon the labyrinthine fluid, whose natural channels of escape are inadequate (during the existence of the normal physiological supply) to establish compensatory egress. Thus, in this instance, it would follow that the compressed *sound conducting fluid* of the labyrinth is immediately responsible for the altered character of certain sounds, the muscle lesion being the causative factor. Owing to the *spiral* nature of the structure in which this compression takes place, the force of impact on the contained fluid becomes less (granted a *moderate* degree of compression) during its ascent, so to speak, toward the apex, on account of its resistance it encounters from *abruptly curved walls.* In other words, a relative inhibition of energy in the molecular impact takes place as a result of continued abnormal propulsion in a spirally-compressed fluid, the increase in pressure causing a deviation from the curvilinear direction *normal to* the fluid contents of the structure.

The reverse would obtain in a conical or a plano-pyramidal structure, for in such a case no *abrupt deflection* would interfere with the necessary augmentation at its distal end of an energy traveling in curved or straight lines. Should any *great* compression, however, exist in any of these structures, supposing each to possess a delicate percipient apparatus such as the *m. basilaris*, its *continuance* would cause a total obliteration of the function of that portion of the apparatus to which the greatest pressure was applied, and a mechanical perversion of function in other portions, varying according to the degree of compression. It would thus seem that the degree of variation for a given tone in the scale will depend upon the degree of compression existing in a certain area in a corresponding turn of the cochlea.

In Case I it will be seen that this hypothesis is borne out in that the highest tones do not show as great a departure from the normal, owing to the greater velocity of the wave impulses and to the situation of the short fibres, as do the lower tones which are perceived in the upper turn of the cochlea and which becomes modified in the manner described. No other hypothesis would explain why in this case the lower tones are heard *lower,* and the higher tones *higher,* in the affected ear; for, although there is an *absolute* increase (in volume) of intra-labyrinthine pressure, the very velocity imparted to the wave-impulses in the lower turn of the cochlea becomes the instrument of their destruction, resulting in an *actual diminution of tension* and a consequent lowering of tone in the upper turn. Further proof of this proposition is seen in the result of the examination of May 10th, all tones having become more nearly normal as a result of diminished pressure."

